# Multidrug-Resistant *Candida auris* Infections in Critically Ill Coronavirus Disease Patients, India, April–July 2020

**DOI:** 10.3201/eid2611.203504

**Published:** 2020-11

**Authors:** Anuradha Chowdhary, Bansidhar Tarai, Ashutosh Singh, Amit Sharma

**Affiliations:** Vallabhbhai Patel Chest Institute of the University of Delhi, New Delhi, India (A. Chowdhary, A. Singh);; Max Health Care Institute, New Delhi (B. Tarai);; International Centre for Genetic Engineering and Biotechnology, New Delhi (A. Sharma)

**Keywords:** 2019 novel coronavirus disease, coronavirus disease, COVID-19, severe acute respiratory syndrome coronavirus 2, SARS-CoV-2, viruses, respiratory infections, zoonoses, Candida auris, antimicrobial resistance, co-infection, nosocomial infection, India

## Abstract

In New Delhi, India, candidemia affected 15 critically ill coronavirus disease patients admitted to an intensive care unit during April–July 2020. *Candida auris* accounted for two thirds of cases; case-fatality rate was high (60%). Hospital-acquired *C. auris* infections in coronavirus disease patients may lead to adverse outcomes and additional strain on healthcare resources.

The ongoing coronavirus disease (COVID-19) pandemic has overwhelmed healthcare systems worldwide. Reports from China and New York have highlighted the concern for nosocomial infections, primarily bacterial, in critically ill COVID-19 patients (*1*–*3*). Secondary *Candida* spp. bloodstream infections in COVID-19 patients with prolonged intensive care unit (ICU) stays have not been documented. However, a new concern coinciding with the brisk expansion of critical care facilities for COVID-19 patients is the potential for nosocomial spread of *Candida auris* infections (*4*). *C. auris* is a global health threat because of its ability to colonize skin, persist in environments, cause nosocomial outbreaks, and lead to severe disease with high mortality rates (*5*,*6*).

## The Study

Following up on our prediction (*4*), we report bloodstream infections caused by multidrug resistant *C. auris* in 1 COVID-19 ICU in New Delhi, India. A total of 596 patients with confirmed COVID were admitted to the 65-bed ICU during April–July 2020. Of these, 420 patients required mechanical ventilation. Overall, candidemia was detected in 15 (2.5%) of the 596 ICU patients; the predominant agent was *C. auris* for 10 (67%) of those patients. For the remaining 5 patients, candidemia was caused by *C. albicans* (n = 3), *C. tropicalis* (n = 1), and *C. krusei* (n = 1).

We abstracted the following data for the candidemia patients: baseline demographics, medical history, laboratory parameters, microbiological findings, concomitant antimicrobial drug use, and treatments. Isolates were identified by matrix-assisted laser desorption/ionization time-of-flight mass spectrometry (MALDI Biotyper, https://www.bruker.com). In addition, species identification was conducted by amplification and sequencing of the internal transcribed spacer region of ribosomal DNA and of the D1/D2 domain of the large subunit ribosomal DNA. Antifungal susceptibility testing was performed by using the Clinical and Laboratory Standards Institute broth-microdilution method M27-A3/S4 (*7*). Antifungals tested were fluconazole, voriconazole, posaconazole, isavuconazole, 5-flucytosine, caspofungin, micafungin, anidulafungin, and amphotericin B.

Most of the 10 patients with *C. auris* infection were elderly (8 patients were 66–88 years of age) and male (7 patients) (Table 1, https://wwwnc.cdc.gov/EID/article/26/11/20-3504-T1.htm). *C. auris* was cultured from paired blood samples from all 10 patients and also from urine for 2 of these patients. All of the COVID-19 patients in whom *C. auris* infections developed had been hospitalized in the ICU for prolonged periods (20–60 days) and had underlying chronic conditions (e.g., hypertension, n = 7; diabetes mellitus, n = 6; and chronic kidney and liver disease, n = 2). Candidemia caused by *C. auris* developed 10–42 days after admission. Half (50%) of the patients with *C. auris* infections received mechanical ventilation as a result of severe COVID-19 pneumonia. Furthermore, all patients with candidemia had indwelling central lines and urinary catheters. Of the 15 patients, COVID-19 was hospital acquired for 2 (acquired 2 and 7 weeks after hospital admission). Severity parameters for COVID-19 were elevated for all patients with candidemia (Table 1). Among the 15 candidemia patients, 8 (53%) died; among those with *C. auris* infection, the fatality rate was 60%. Of note, 4 of the 6 patients who died experienced persistent fungemia, and despite micafungin therapy for 5 days, *C. auris* again grew in blood culture.

**Table 1:** Analytical findings for 15 coronavirus disease patients with candidemia, India, April–July 2020*

Patient no.	1	2	3	4	5	6	7	8	9	10	11	12	13	14	15
Age, y/sex	25/F	52/M	82/F	86/F	66/M	71/M	67/M	72/M	81/M	69/M	56/M	69/M	43/F	47/M	66/M
Days of hospitalization	35	20	60	21	20	32	21	27	20	21	18	27	24	18	28
Day of SARS-CoV-2 positivity	1	1	41	1	1	1	1	1	1	1	1	7	1	12	1
Day(s) of blood culture *Candida spp.* positivity	14 (*Candidia auris*)	14 and 17 (*C. auris*)	42† and 47 (*C. auris*)	10 (*C. auris*)	11 and 15 (*C. auris*)	12† and 17 (*C. auris*)	11 (*C. auris*)	16 and 19 (*C. auris*)	15 (*C. auris*)	14 (*C. auris*)	7 (*C. tropicalis*)	8 (*C. albicans*)	12 (*C. albicans*)	5 (*C. albicans*)	7 (*C. krusei*)
Risk factors	CLD with grade II hepatic encephalopathy, AKI	HT, DM	HT, DM, hypothoidism, on dialysis for CKD stage 5	CLD, IHD, DM	HT, DM, asthma	Hypothoidism, on dialysis for CKD stage 5	HT, DM, COPD	HT, CLD	DM, HT, IHD	HT, asthma	HT, COPD	HT, DM, obesity, IHD	HT	Asthma, DM	HT
Laboratory parameters on day of candidemia diagnosis (reference range)															
Lymphocytes, × 10^9^ cells/L (1–3)	0.4	1.2	0.7	0.8	1.2	0.7	0.4	1.2	0.6	1.2	0.6	1.1	0.4	0.9	1.2
Total leucocytes, × 10^9^ cells/L (4–10)	5.8	15.3	18.2	15.8	15.3	18.2	17.2	19.8	12.8	7.8	21.2	15.3	17.8	14.2	12.3
Thrombocytes, × 10^9^ cells/L (150–410)	75	170	45	170	170	45	142	160	160	200	221	164	120	110	124
Hemoglobin, g/dL	6.2	10.2	6.7	11	10.2	6.7	11.3	12.5	9.8	12.1	9.8	10.2	9.7	10.2	11.8
D dimer, ng/mL (0–243)	1,068	3,024	4,319	980	2,973	3,024	670	560	480	760	1,002	778	278	543	670
LDH, U/L(<247)	428	780	668	298	780	668	652	927	667	778	668	348	447	343	924
Ferritin, ng/mL (11–306.8)	626.6	993.1	1743	405.6	528.7	1,624	980	448	408.7	574.5	876	985	765	678.2	348.7
Procalcitonin, ng/mL (<0.5)	10.71	20.23	5.73	102	30.8	110	5.82	102	5.75	8.76	128.2	110	9.8	10.7	24.2
CRP, mg/dL (<1)	4.2	9.2	14.6	3.2	9.2	14.6	9.5	14.6	8.3	5.8	9.1	4.2	3.2	12.8	14..5
Urea, mg/dL (17–43)	150	54	253	58	44	62	38	48	108	34	54	42	22	44	53
Creatinine, mg/dL (0.6–1.1)	28.7	2.3	16.5	21.2	2.7	15.6	4.8	15.3	2.1	1.8	3.4	7.8	6.5	7.8	21.2
Ammonia, μg/dL (10–80)	200	78	132	138	82	121	110.2	131	73	52	84	134	87.9	124	132
Aspartate aminotransferase, U/L (<50)	82	58	54	82	58	54	51	57	63	58	62	72	54	62	58
Alanine aminotransferase, U/L (<35)	38	28	10	38	28	10	7	9	38	27	10	26	8	10	9
Alkaline phosphatase, IU/L (30–120)	161	48	268	161	48	268	116	70	140	120	46	160	46	110	72
Clinical parameters															
Therapy for SARS-CoV-2	AZI, HCQ,	AZI, HCQ, convalescent plasma, TCZ and RDV	AZI, HCQ, FPV	AZI, HCQ, RDV	AZI, RDV	AZI, FPV	AZI, RDV, convalescent plasma	AZI, FPV, TCZ, convalescent plasma	AZI, FPV	AZI, FPV, TCZ, convalescent plasma	AZI, FPV	AZI, FPV,	AZI, RDV	AZI, RDV	AZI, FPV, Convalescent plasma
Antibiotic therapy	TZP, TEC, MEM	TZP, TEC	TZP, DOX, PMB	TZP, MEM	CRO, DOX	TZP, DOX, PMB	CFM, MEM	AMC	TZP, MEM	CFM, MEM	AMC	TZP, DOX,	CFM, MEM	TZP, DOX,	TZP, DOX
Steroid therapy for pneumonia	No	Yes	Yes	Yes	No	Yes	Yes	Yes	Yes	Yes	No	No	Yes	No	Yes
Oxygen support	Ambient air	IMV	IMV	High-flow oxygen	High-flow oxygen	IMV	High-flow oxygen	IMV	High-flow oxygen	IMV	High-flow oxygen	IMV	High-flow oxygen	IMV	IMV
Antifungal therapy	AMB	MFG and AMB	MFG	MFG	MFG and AMB	MFG	AMB and MFG	MFG	MFG	MFG	MFG	MFG	MFG	AMB and MFG	AMB
Outcome	Survived	Died	Died	Died	Survived	Died	Survived	Died	Died	Survived	Survived	Survived	Survived	Died	Died

*AKI, acute kidney disease; AMB, amphotericin B; AMC, amoxycillin/clavulanate; AZI; azithromycin; CFM, cefixime; CKD, chronic kidney disease; CLD, chronic liver disease; COPD, chronic obstructive pulmonary disease; CRO, ceftriaxone; DM, diabetes mellitus; DOX, doxycycline; FPV, favipiravir; HT,hypertension; IHD, ischemic heart disease; HCQ, hydroxychloroquine; IMV, invasive mechanical ventilation; MEM, meropenem; MFG, micafungin; PMB, polymyxin B; RDV, remdesivir; SARS-CoV-2, severe acute respiratory syndrome coronavirus 2; TEC, teicoplanin; TCZ, tocilizumab and remdesivir;, TZP, piperacillin/tazobactum. †*C. auris* isolated from blood and urine*.*

Antifungal susceptibility testing data for *C. auris* isolates from 10 patients showed that all isolates were resistant to fluconazole (MIC >32 mg/L) and 30% were nonsusceptible to voriconazole (MIC >2 mg/L). Furthermore, 40% were resistant to amphotericin B (MIC >2 mg/L) and 60% were resistant to 5-flucytosine (MIC >32 mg/L). Overall, 30% of *C. auris* isolates were multiazole (fluconazole + voriconazole) resistant; whereas, 70% were multidrug resistant, including 30% (n = 3) that were resistant to 3 classes of drugs (azoles + amphotericin B + 5-flucytosine) and 4 that were resistant to 2 classes of drugs (azoles + 5-flucytosine and azoles + amphotericin B). All isolates were susceptible to echinocandins (Table 2, https://wwwnc.cdc.gov/EID/article/26/11/20-3504-T2.htm).

**Table 2:** Drug MICs for *Candida* spp.in 15 coronavirus disease patients, India, April–July 2020*

Drug	Patient no. (*Candida* sp.), drug MIC, μg/mL														
	1 (*C. auris*)	2 (*C. auris*)	3 (*C. auris*) †	4 (*C. auris*)	5 (*C. auris*)	6 (*C. auris*) †	7 (*C. auris*)	8 (*C. auris*)	9 (*C. auris*)	10 (*C. auris*)	11 (*C. tropicalis*)	12 (*C. albicans*)	13 (*C. albicans*)	14 (*C. albicans*)	15 (*C. krusei*)
FLU	512	256	256/256	512	256	256/256	512	256	256	512	0.25	0.5	0.5	0.5	16
VRC	2	0.5	1/1	1	1	1/0.5	0.25	2	2	0.125	0.03	0.125	0.125	0.125	0.125
ISA	0.5	0.25	0.125/0.25	0.03	0.06	0.125/0.25	0.5	0.06	0.06	0.06	0.015	0.125	0.125	0.125	0.125
POS	0.25	0.125	0.125/0.125	0.015	0.06	0.03/0.03	0.125	0.06	0.03	0.06	0.015	0.125	0.25	0.25	0.25
AMB	0.5	0.5	0.125/0.25	0.5	2	1/1	2	4	1	4	0.5	0.5	0.5	0.5	0.5
CAS	2	2	2/4	1	0.5	0.5/0.25	0.25	1	0.5	1	1	0.125	0.125	0.125	10.5
AFG	0.125	0.25	0.25/0.5	0.125	0.25	0.125/0.25	0.5	0.125	0.125	0.25	0.06	0.06	0.03	0.03	0.125
MFG	0.06	0.25	0.5/0.5	0.06	0.03	0.25/0.125	0.125	0.25	0.125	0.125	0.06	0.06	0.06	0.06	0.125
5-FC	32	8	8/16	1	1	64/64	64	64	64	64	0.125	0.5	1	1	16

*AFG, anidulafungin; AMB, amphotericin B; CAS, caspofungin; FLC, fluconazole; ISA, isavuconazole; MFG, micafungin; POS, posaconazole; VRC, voriconazole; 5-FC, 5-flucytosine. †*C. auris* isolated from blood and urine.

## Conclusions

Our findings highlight the role of hospital-acquired *C. auris* bloodstream infections; the patients were probably infected while hospitalized. *C. auris* can be transmitted in healthcare settings just like other multidrug-resistant organisms, such as carbapenem-resistant *Enterobacteriaceae* and methicillin-resistant *Staphylococcus aureus* (*4*). For 4 of 10 patients studied, bacteremia caused by *Enterobacter cloacae* and *Staphylococcus haemolyticus* was also noted. In patients with severe COVID-19, the rate of secondary infections was substantially higher, as has been reported by Goyal et al. (6% of cases of secondary bacterial infections in the United States) (*3*) and Zhou et al. (15% of cases of secondary bacterial infections in China) (*8*). Among fungal co-infections in France, the incidence of putative invasive pulmonary aspergillosis was high (30%) (*9*).

Several major outbreaks of bloodstream infections caused by *C. auris* have been reported in India, the United Kingdom, Colombia, South Africa, and the United States (*5**,**10**–**12*). In our report, all patients in the ICU had indwelling invasive devices such as central venous and urinary catheters, which may be the source of *C. auris* infections (i.e., candidemia and urinary tract infection). We anticipate that transmission of *C. auris* to COVID-19 patients by healthcare personnel is unlikely because of the use of personal protective equipment. However, incorrect and extended use of personal protective equipment can lead to self-contamination and transmission. 

Of note, 6 of the 10 patients died, possibly because of multiple underlying health conditions. However, 67% of those who died had persistent candidemia, which may have contributed to their death. Furthermore, multidrug-resistant *C. auris* affects the choice of antifungal therapy and treatment outcomes. Most *C. auris* isolates are resistant to fluconazole, and panresistant isolates have been described (*13*). All *C. auris* isolates in our study were resistant to fluconazole, and 40% were resistant to amphotericin B, both of which are commonly used in resource-limited countries; therefore, resistance to both classes of drug by *C. auris* is highly concerning because use of other antifungals such as echinocandins are limited in these countries.

Candidemia affected 2.5% of the COVID-19 patients in this cohort admitted to the ICU. In a tertiary care center in New Delhi, *C. auris* was reportedly the second most common *Candida* species that caused candidemia in non-COVID patients (*14*). Extensive contamination of the hospital environment has been detected in hospitals experiencing outbreaks of *C. auris* infection*,* warranting adherence to strict hospital infection prevention practices, such as enhanced cleaning of rooms with chlorine-based disinfectants at high concentrations (0.5%) for highly resistant pathogens such as *C. auris*. Critically ill COVID-19 patients with *C. auris* infection tend to have concurrent conditions (e.g., diabetes mellitus, chronic kidney disease) and risk factors (e.g., need for mechanical ventilation, receipt of steroids). To reduce complications, admission times in overburdened hospitals, and death rates among COVID-19 patients, identifying and treating *C. auris* infections is vital. A recent report that investigated changes in the fecal fungal microbiomes of COVID-19 patients has shown increasing prevalence of opportunistic fungal pathogens such as *C. albicans*, *C. auris*, and *Aspergillus flavus* (*15*). These data, along with our findings, provide evidence that the ongoing COVID-19 pandemic may provide ideal conditions for outbreaks of *C. auris* in hospital ICUs (*4*). Thus, during the COVID-19 pandemic, extra caution is warranted in hospitals, regions, cities, and countries where *C. auris* is prevalent.
